# Diseases of the Nerves, Muscles and Skin

**Published:** 1887-01

**Authors:** 


**Diseases of the Nerves, Muscles and Skin,** being Vol. III. of Dr. Hermann Eichhorst's Handbook of Practical Medicine, and Vol. X. of Wood's Library of Standard Medical Authors, 1886 (consisting of 12 vols., price, $15.00). Sold only by subscription. New York: William Wood & Company.

